# Residents’ experiences during a hydrogen sulfide crisis in Carson, California

**DOI:** 10.1186/s12940-024-01071-5

**Published:** 2024-03-23

**Authors:** Arbor J. L. Quist, April Hovav, Alexander D. Silverman, Bhavna Shamasunder, Jill E. Johnston

**Affiliations:** 1 Department of Population and Public Health Sciences, Keck School of Medicine, University of Southern California, 1845 N Soto St., Los Angeles, CA 90032 USA; 2 Department of Urban & Environmental Policy, Occidental College, Los Angeles, CA USA

**Keywords:** Environmental justice, Disaster, Emergency response, Odors, Perceptions, Environmental health

## Abstract

**Background:**

In early October 2021, thousands of residents in Carson, California began complaining of malodors and headaches. Hydrogen sulfide (H_2_S), a noxious odorous gas, was measured at concentrations up to 7000 parts per billion (ppb) and remained above California’s acute air quality standard of 30 ppb for a month. Intermittent elevations of H_2_S continued for 3 months. After 2 months of malodor in this environmental justice community, a government agency attributed the H_2_S to environmental pollution from a warehouse fire. Research has yielded conflicting results on the health effects of H_2_S exposure at levels that were experienced during this event. This research fills a critical need for understanding how people perceive and experience emergent environmental health events and will help shape future responses.

**Methods:**

Through a community-academic partnership, we conducted 6 focus groups with 33 participants who resided in the Carson area during the crisis. We sought to understand how this incident affected residents through facilitated discussion on topics including information acquisition, impressions of the emergency response, health symptoms, and ongoing impacts.

**Results:**

The majority of participants were women (*n* = 25), identified as Latina/o (*n* = 19), and rent their homes (*n* = 21). Participants described difficulty obtaining coherent information about the emergency, which resulted in feelings of abandonment. Most participants felt that local government and healthcare providers downplayed and/or disregarded their concerns despite ongoing odors and health symptoms. Participants described experiencing stress from the odors’ unknown health effects and continued fear of future odor incidents. Residents sought to take control of the crisis through information sharing, community networking, and activism. Participants experienced longer term effects from this event, including increased awareness of pollution and reduced trust in local agencies.

**Discussion:**

This study demonstrates the necessity of clear, comprehensive, and prompt responses by relevant decisionmakers to chemical emergencies to appropriately address residents’ fears, curb the spread of misinformation, and minimize adverse health effects. Participant responses also point to the benefit of supporting horizontal community networks for improved information sharing. By engaging directly with community members, researchers and disaster responders can better understand the various and complex impacts of chemical disasters and can improve response.

**Supplementary Information:**

The online version contains supplementary material available at 10.1186/s12940-024-01071-5.

## Introduction

**Fig. 1:**
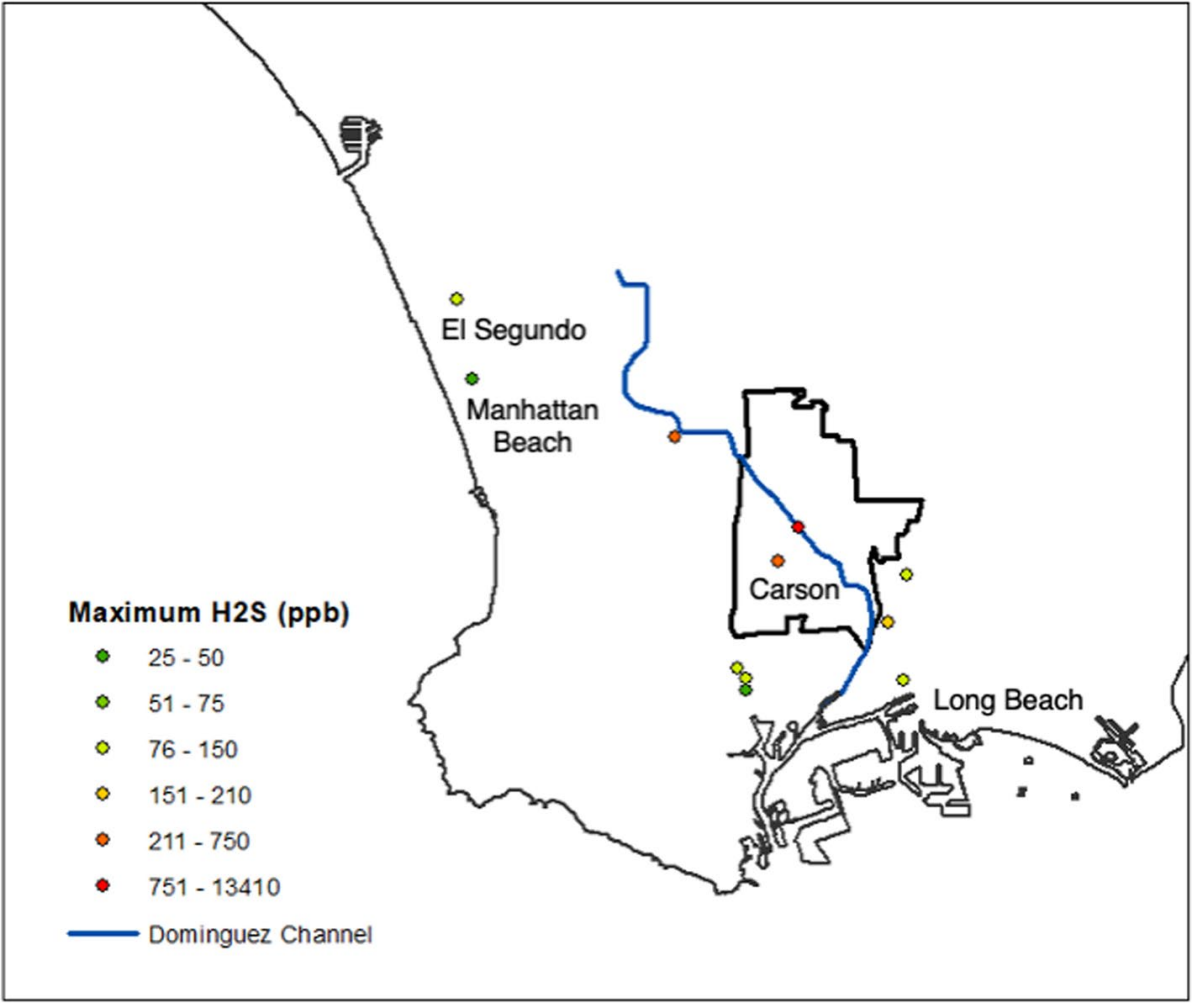
Map of the South Bay of Los Angeles, California and maximum 5-min hydrogen sulfide concentrations measured at South Coast Air Quality Management District’s stationary monitors in October 2021 [11]

Residents in the South Bay of Los Angeles (LA) County, California abruptly started to complain of malodors, nausea, dizziness, and headaches in early October 2021 [1]. The South Coast Air Quality Management District (SCAQMD) identified the odor as hydrogen sulfide (H_2_S), a toxic odorous gas known for its characteristic rotten-egg smell. H_2_S concentrations peaked at 7000 parts per billion (ppb) in Carson, California and remained above California’s one-hour average air quality standard of 30 ppb for 4 continuous weeks, with intermittent elevations continuing for the next 3 months [2]. H_2_S concentrations were highest along the Dominguez Channel in Carson (Fig. 1), but the cause of this H_2_S crisis was not identified for 2 months when officials declared the emergency was tied to a warehouse fire in late September 2021 [3, 4]. In extinguishing the fire, ethanol, isopropyl alcohol, and benzene were released into the channel, which led to the anaerobic decay of materials in the channel and produced large amounts of H_2_S. By the end of November 2021, the County of LA had distributed 40,000 indoor air purifiers, provided 3400 hotel rooms, and spent $5.4 million applying a biodegradable odor neutralizer to the Channel; however, many residents were unsatisfied with the response as there was limited information about the cause or consequences of the event [3]. Information on this H_2_S event was available on the SCAQMD website, LA County Department of Public Health website, City of Carson website, and in the news [5–7]. LA County Public Works posted on Twitter in mid-October 2021 that residents in Carson were experiencing rotten egg odors from hydrogen sulfide and included a phone number to call for resources and information [8]. Aside from social media, websites, and the news, information was not systematically distributed throughout the Carson area; however, the County of Los Angeles hosted a virtual community meeting with officials from SCAQMD, LA Department of Public Health, and LA County Public Works to answer questions about the odors and the continuing investigation in fall 2021 [9, 10].

Carson, California is a highly diverse city that is heavily burdened with oil refineries, industrial facilities, and freeways polluting the environment [12]. The California Communities Environmental Health Screening Tool (CalEnviroScreen) has ranked Carson in the top quartile of statewide pollution burden; CalEnviroScreen incorporates both environmental exposures and population characteristics, highlighting Carson as a community with both high exposure to pollution and sensitive populations vulnerable to contamination [12]. Carson has also contended with a history of systemic racism. For example, the Dominguez Channel—which runs through the City of Carson—was previously known by a racist name [13]. Residents in east Carson, on the border of Long Beach, experienced these noxious odors in addition to their regular exposure to high levels of toxic releases and particulate matter and other chemicals from a nearby large refinery [12]. Throughout Southern California and across the United States, environmental contaminants are unequally distributed, with communities of color and low-income communities disproportionally exposed [14, 15]. Existing environmental injustices and historical inequalities frequently influence disaster vulnerabilities [16].

Prior research on the health effects of the H_2_S exposure at concentrations that were experienced during this emergency have yielded conflicting results [17–24]. We found the H_2_S crisis associated with an increase in rates of emergency department visits for asthma, acute upper respiratory infections, headaches and migraines, and dizziness [25]. However, there remains a gap in understanding how people perceive and experience odor emergencies, including the H_2_S event in Carson. Filling this research gap will help drive future policy and responses during environmental health emergencies and has the potential to improve communication on hazards and recommended actions, increase trust between residents and regulatory agencies, and reduce residents’ stress, uncertainties, and adverse health effects during environmental crises.

This H_2_S crisis interrupted daily lives due to the stench and health symptoms. Lack of personal control over events, conflicting information about exposures, and institutional delegitimization contribute to stress during a contamination event, which can harm victims’ mental and physical health [26–29]. In addition to understanding how environmental toxins affect human health, qualitative data collection can help us consider how contamination may be interpreted by residents and how residents deal with these traumatic threats to their health and safety. Qualitative research methods are useful in understanding residents’ experiences and perceptions during an environmental disaster and can increase our understanding of complex exposure pathways, including how social factors contribute to environmental health symptoms during a disaster [30]. In the environmental justice movement, stories play an important role in sharing experiences and building support for grassroots activism [14].

Odors are particularly difficult to measure, making qualitative research and residents’ experiences especially important. For example, some citizen scientists use residents’ detailed local knowledge of the environment and odor complaint data as environmental monitoring tools [31]. Several studies have used daily diaries and have found malodors associated with stress, sneezing, runny nose, alternation of daily activities, negative mood states, and decreased sleep duration [32, 33]. In-depth interviews have also highlighted how odors limit daily activities of nearby residents, including cookouts, family reunions, socializing with neighbors, gardening, playing, drying laundry outside, and opening windows for fresh air [34–36]. Additionally, interviews have identified the frustration and injustice that many residents feel when consistently exposed to malodors and when receiving ambiguous statements from officials regarding the odors’ health effects [34]. Surveys have been used to associate malodors and hydrogen sulfide with various health symptoms; several studies have found odors to mediate the relationship between industrial exposures and health symptoms [21, 37–39]. However, literature is mixed on the effects of low-level H_2_S exposure; some studies have found no association between odors and self-reported health, objective physical symptoms, and mood [40, 41]. Much of the odor literature focuses on landfills and industrial livestock farming, and thus is typically conducted in relatively rural areas [32–34, 36]. More research is needed on how malodors impact urban environmental justice neighborhoods.

Malodors are typically regulated as nuisances; however, nuisances can be difficult to regulate by state and federal pollution laws [42, 43]. Nuisance-based odor regulations that are overseen by regional air quality management districts are often ineffective because they depend on inspectors to confirm odor complaints, and odors often dissipate by the time inspectors arrive [43]. In California, H_2_S is regulated as a nuisance at 30 ppb rather than based on health protective standards; [44] additional research is needed to understand the health effects of episodic and subacute H_2_S exposure. Nevertheless, scientists have expressed that distinctions between regulatory responses to odor pollution being regulated on nuisance/annoyance vs. health effects is based on legal and not scientific interpretations [45].

In the aftermath of the 2021 odor crisis in Carson, we worked with community partners to launch a rapid health survey. The majority of survey respondents indicated that the malodor had affected their physical and mental health, with headaches and dizziness being the most commonly reported symptoms [46]. To expand upon and contextualize these results, we conducted focus groups to further understand the various degrees in which residents were impacted during this H_2_S emergency.

## Methods

### Designing focus group guide

Our academic team developed the initial draft of the focus group guide, which included discussion questions on information acquisition during the event, feelings about government response, potential immediate and lasting impacts of the incident, and thoughts about community and the environment. A community partner gave feedback on the guide and topics, after which we finalized the guide (see Supplementary Materials). Once finalized, a bilingual staff member and a native Spanish-speaking staff member translated the guide into Spanish.

### Conducting focus groups

We recruited focus group participants from an existing list of study participants who completed a health survey on the Carson odor crisis. These participants were originally recruited for the health survey by working with community groups, talking to residents on the street, going door-to-door, and posting in a Carson Facebook group. Study staff telephoned and/or sent text messages to survey participants who indicated interest in participating in a focus group. We attempted to recruit 5–8 participants for each focus group.

We conducted 6 focus groups with a total of 33 participants to understand the complex ways in which this H_2_S crisis affected residents. Focus groups were held between September 2022 and March 2023, with each focus group lasting 1–2 h. Focus group participants were given $40 gift cards for participating. Four focus groups were held in English and 2 were conducted in Spanish. This study was approved by the University of Southern California Institutional Review Board; the study was described to all participants, and all participants read and signed informed consent forms. One interviewer led the majority of the discussion, with another interviewer sitting in to ask occasional probing questions.

### Analyzing data

Focus groups were recorded with the permission of the participants, and all focus groups were transcribed in their original language. Participant IDs were used in the transcripts instead of names. The transcriptions were uploaded to Atlas.ti Web and were coded based on focus group guide themes and emergent themes. Two researchers developed an initial codebook based on a discussion of themes observed in the focus groups. Two researchers coded each focus group; researchers discussed differences in coding until an agreement was reached. New codes were iteratively added, as needed. We identified main themes and then shared them with five focus group participants from various focus groups in order for participants to confirm main themes and/or add themes that academic researchers may have missed.

## Results

**Fig. 2:**
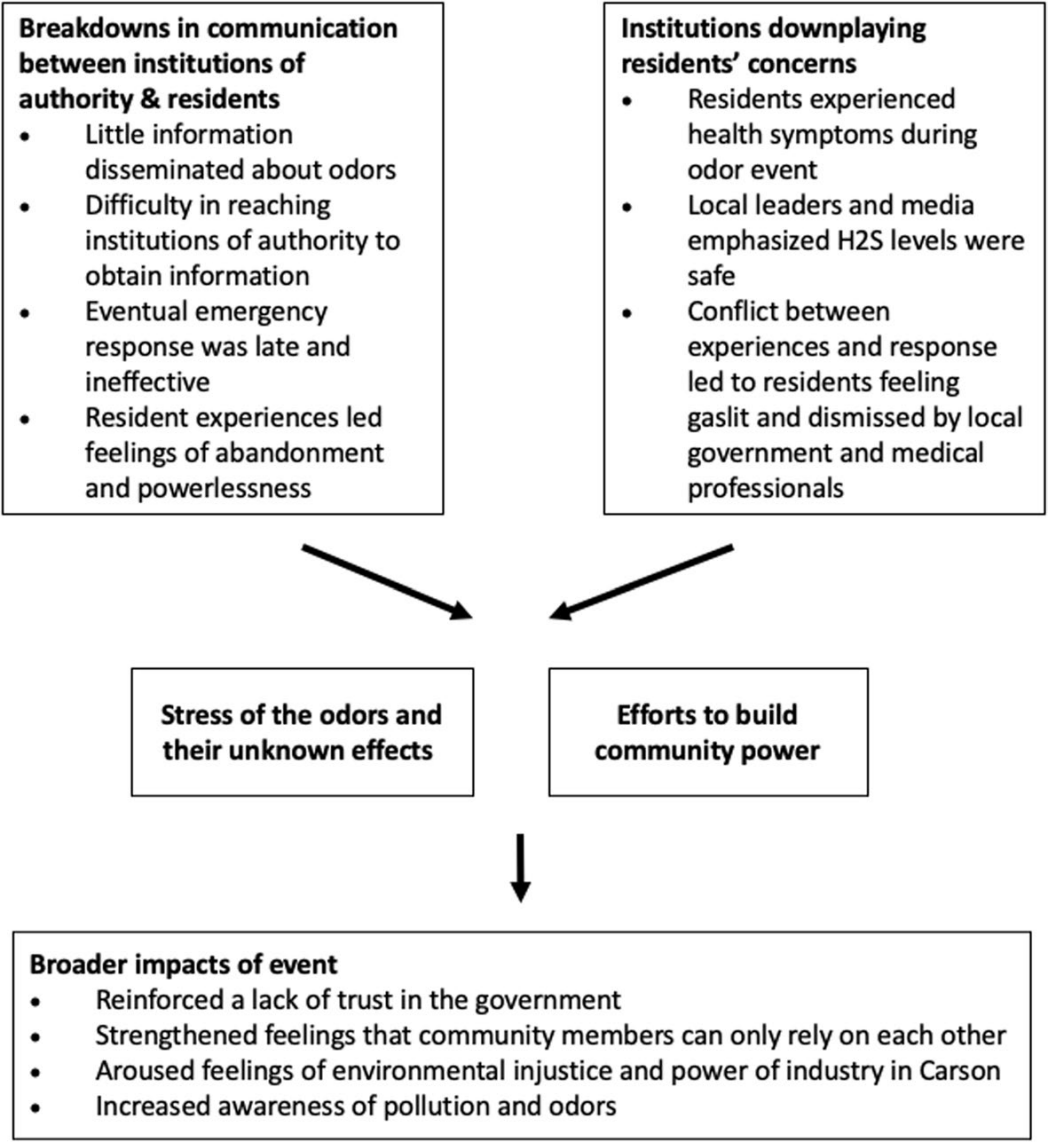
Conceptual framework of themes and subthemes identified in focus groups

Of the 33 participants in the six focus groups, 25 were women and were 8 were men. The majority identified as Latino/Hispanic (*n* = 19), with Black (*n* = 9), white (*n* = 4), Native American (*n* = 1), and Asian (*n* = 1) identities also represented. Participants ranged in age from 18 and 74 years. The majority of participants rented their homes (*n* = 21) and have lived in the community > 5 years (*n* = 21). Most participants lived within 2 miles of the Dominguez Channel (*n* = 19), with only 3 participants living farther than 3 miles from the Channel (Table 1). From these focus groups, we identified five main themes, 1) lapse in communication by decision makers and associated feelings of abandonment, 2) local leaders downplaying residents’ concerns which led to residents feeling gaslit, 3) efforts to build power through community networks, activism, and self-research, 4) stress of the odors and their unknown health effects, and 5) long-term impacts of the event, including increased awareness of pollution and odors (Fig. 2).

**Table 1:** Demographic characteristics of study participants

**Characteristic**	**n (%)**
Total	33 (100%)
Age	
18–30	6 (18%)
31–45	8 (24%)
46–65	13 (39%)
> 65	6 (18%)
Female	25 (76%)
Language of focus group	
English	19 (58%)
Spanish	14 (42%)
Race/ethnicity	
Asian	1 (3%)
Native American	1 (3%)
Latino/Hispanic	19 (58%)
Black	9 (27%)
White	4 (12%)
Education	
Grade 8 or less	5 (15%)
Some high school	2 (6%)
High school graduate	3 (9%)
Some university/college	10 (30%)
Associates degree or technical school	3 (9%)
Bachelor’s degree	5 (15%)
Graduate degree	3 (9%)
Employment	
Homemaker	10 (30%)
Employed outside the home	14 (42%)
Retired	7 (21%)
Student	2 (6%)
Home type	
Apartment	9 (27%)
Condo/townhouse	3 (9%)
Single-family house	18 (55%)
Mobile home	2 (6%)
House ownership	
Own home	11 (33%)
Rent home	21 (64%)
Distance from Dominguez Channel	
≤ 1 mile	5
> 1–2 miles	14
> 2–3 miles	11
> 3–8 miles	3

### 1) “We didn’t hear anything:” Breakdowns in communication between institutions of authority and residents

#### Residents received little information about the odors

Participants described being confused by the source of the odors and receiving little or no information about the odors from responsible agencies. Residents were bewildered by the situation and sensed that there was a problem because of the combination of odors and health symptoms. One Black woman described:

“We could smell it. And didn’t have a clue as to what it was really. We didn’t understand what was going on.” (Focus group 1, participant 1)

Several participants expressed frustration that they did not receive an immediate notification about the odors from the local government because “we all have the right to know what is happening in our community” (FG5 P1; translated from Spanish, original Spanish quotes in Supplementary Materials). A White participant expressed, “we didn’t hear anything from the government,” and most participants agreed (FG4 P5). A few participants thought that the odors were an individual issue just occurring on their property because of the lack of information. In fact, one participant moved to a new city because of the odor, which he had not realized was a community-wide issue.

#### Residents struggled to reach local government to obtain information

Even when residents sought information by calling public officials, they found it difficult to reach someone on the phone to obtain answers. Many participants explained that the lines were consistently busy, and their calls and messages were seldom returned. One Latina described, “I called a few different times to the number they gave me when I came here [HOA meeting] and I kept calling but they never answered” (FG5 P5, translated from Spanish). Only one participant said they were satisfied with the information they received when they called the City of Carson. Participants also expressed uncertainty around who was leading the response, which made it confusing for them to know whom to contact. As participants described their interactions with and disappointment in the responsible agencies, participants referred to the perceived responsible agency as “they” (e.g., “they didn’t give us information”); however, participants were often referring to multiple agencies (e.g., the City of Carson vs. South Coast Air Quality Management District vs. LA County Department of Public Works).

#### Residents worried they were receiving misinformation

Since most people got information from unofficial sources, some people worried about misinformation. One woman explained,

There were other people that are just regular citizens that were really putting in the information and making it possible. And it was scary, because you didn’t know what was a rumor or what was true. You know we didn’t know if we were really going to be reimbursed [for our hotel]. (FG2 P3)

Misinformation, or lack of information, could also have resulted in dissuading residents from utilizing resources that may have been available to them. Primarily Spanish-speaking participants were under the impression that to be eligible for an air purifier “they have to be the people that live right there, right on the bank of the channel” (FG5 P6, translated from Spanish) even though their addresses would have qualified them to receive a purifier (although this was confusing because the LA County Department of Public Works changed the area in which residents were eligible for an air purifier during the event, and many residents were unaware of the change in the eligibility criteria). The dearth of official information was tied to the spread of rumors. One person expressed, “No one is spreading misinformation intentionally, but we’re not hearing from anyone officially, so we don’t know” (FG2 P3).

#### Residents felt that the eventual response was late and insufficient

Most participants expressed that the response to the odors was very delayed and ineffective. One Black resident noted, “the city waited too late to let the community know what’s going on after they knew what happened” (FG4 P4). Participants felt like it took “about a month or two” to get information about the odors and to obtain air purifiers, but even then “the lines [for the air purifiers] were horrendously long” (FG2 P2).

#### Difficulty in obtaining clear information about the odors led to feelings of abandonment and powerlessness

Most participants believed the odors were harmful to them and felt “disposable”, “disregarded”, and “completely abandoned” (FG2 P2) when they perceived little was being done to address the issue. A Black man expressed that he expected a better response from his local leaders:

You’re paying taxes and you live in a community, and you know, you’re pretty much proud of your community that you live in. You try to do what you do to stay active and be active in your community and for people that you entrust and vote in office to just shut you down and not answer you and just act like you don’t exist. That’s pretty frustrating. (FG1 P2)

Participants who live on the east side of Carson near Long Beach expressed that they were particularly ignored because they live in a “forgotten city” with “less resources” and little accountability (FG5 P6, translated from Spanish). A Latina participant explained,

“And because our postal code shows up as Long Beach, they tell us we are a part of Long Beach and not Carson, and that’s why we don’t get any resources. It is something that we have been fighting against....When they beautify Carson, they improve the Avalon area. All of that area is improved, but this part, the forgotten part, they barely gave us a crossing guard and all that” (FG5 P7, translated from Spanish).

Residents in these areas described that during the odor event, “we don’t know who to go to. Maybe because we are farther from the center of Carson over there” (FG6 P1, translated from Spanish). One Latina participant explained, "We have not had any support, no help, nothing” (FG5 P5, translated from Spanish).

Spanish-speaking participants in particular described feelings of powerlessness and the necessity of adapting. A Latino young adult expressed, “I feel like we’re just forced to deal with it” (FG4 P8). A Latina participant explained:

You tell yourself, it’s not in my hands to solve the problem, so you just have to learn how to live with it. You can’t do anything because your job is right there, and even if you are suffocating and you don’t like how you are feeling, you have to continue with your work you can’t just quit your job. So that’s why I think we had to adapt to the situation (FG6 P2, translated from Spanish).

### 2) “You know that something is wrong. And you’re being told that it’s not happening:” Institutions downplaying residents’ concerns

#### Residents experienced health symptoms during the odor event

Most focus group participants described experiencing respiratory issues and “really, really bad headaches” (FG1 P5), with several participants reporting dizziness, trouble sleeping, nausea, gastrointestinal issues, and one participant describing passing out. Several participants expressed that their symptoms were severe, and a few explained that they “couldn’t breathe” and went to urgent care (FG4 P3).

#### Local leaders and media emphasized that H_2_S levels were not harmful

While participants were experiencing health symptoms that they associated with the odors, they described that they were simultaneously hearing from the media and government officials that the odors would not cause health effects. A Black woman explained that she was told that the odors were “not a big deal…the levels aren’t that high…[and are] not harmful in any way, shape, or form” (FG1 P1).

#### Conflict between experiences and response led to residents feeling dismissed

The information that participants received from official channels seemed to clash with their own experience of the event, leading residents to feel gaslit. Participants discussed personally experiencing headaches and respiratory issues while hearing information that the odors have no effect on health:

I think what upsets me was that every time they talked about it on the news, they were like, oh, but it’s not a health effect. You’re going to be fine. You don’t have to worry about it being something to affect your health and safety. It’s just a bad smell. But I don’t think we were realizing that people were getting headaches and bloody noses. It was much more than they were making it seem. And that’s why I felt like our information from the group was more reliable than what we were getting from the news. Everything I was watching on the news, I was like, they’re just kind of blowing it off. They’re kind of making it seem like it’s nothing, but it really wasn’t. (FG4 P1)

Participants suffering health effects said they were left to wonder, “am I exaggerating this?” (FG3 P1). Most participants expressed frustration in how they felt that local leaders, the media, healthcare providers, and responsible agencies were downplaying their concerns and health symptoms. As one Black woman described:

It is very disheartening to not be heard. And you know that something is wrong. And you’re being told that it’s not happening. And then you have a collective group of other people who are experiencing the same things. And we’re being collectively told that it’s not happening….This group collectively was seen as, to me, a group to disregard. It was gaslighting to the highest level, and it was a total insult to our intelligence because we could smell it. Stop telling me that it’s this diminished issue when we’re getting sick. People are getting sick, and it’s going on and on…to this day I still don’t think they’ve called it poisoning. We were poisoned, and they did not acknowledge it as such. They kept calling it a noxious odor and a smell. (FG2 P3)

These sentiments are representative of what we heard from many participants. In addition to feeling disregarded by local government and media, several participants who sought help from medical professionals or veterinarians also felt that their concerns were dismissed and ignored. One woman stated:

Veterinarians and doctors were not aware of what was going on in Carson. And it was almost kind of broaching a foreign subject with them. I at one point actually felt like I was being gaslighted because their reactions were they hadn't heard about anything. (FG3P1)

This conflict between the perceived lack of response and lived experiences led to irritation and decreased trust in responsible agencies.

### 3) “It’s the unknown….it’s not knowing how it’s affecting my health”: Stress of the unknown impacts of the odors on health

#### Stress during the odor crisis

Most participants expressed frustration due to the uncertainty regarding what caused the odors and confusion and fear of the unknown health effects of the odors. Although many residents experienced health symptoms, they did not know how the odors were related to their health. Several participants noted that the unknowns related to the odors caused stress. One Latino participant explained,

I don’t know where these smells are coming from, and it’s not knowing how it’s affecting my health. And that concern and knowing that, like the areas around this industry…Knowing all that information is kind of stressful, especially when you’re reminded that there’s a smell in the area, by just going outside and trying to take a deep breath. (FG2 P1)

Participants noted that the odors themselves, the information about the odors, the lack of information, and the unknown effects of the odors affected their stress. One woman expressed,

This is like been one of the most kind of frustrating, anxiety-producing situations I've ever run into....I would say that my health has actually suffered because of the stress. I developed blood pressure issues. (FG3 P1)

#### Stress and fear after the odor crisis

Participants also described anxiety and fears of future odor emergencies since they “never know when it’s going to happen again” (FG4 P6). A few participants felt certain that the odors would return, and they expressed living with this continual fear and stress. Although many participants agreed that the worst of the odors had subsided, a Latina participant explained how the event continues to impact her: “it’s a mental thing now” (FG4 P1). Focus group participants also talked about how they still view the places in their community where the odors were especially bad as dirty and contaminated places. A Black woman discussed how the event continues to affect her family and community:

The kids play outside and everything, but nobody feels very secure here, and it’s been a year, and so sometimes I’ll get a headache, or the kids will get a headache, and we just wonder, is that still it? Because those issues were never a problem prior to this event. (FG2 P3)

Participants also described feeling stress and increased awareness of odors because “you don’t know how it’s going to affect you in the long run” (FG4 P4). Another woman expressed the fear associated with this unknown:

“And that is the fear of being a Carson resident, that maybe in this moment we don’t have any condition, or any sickness. But maybe in the future you can develop lung cancer or something that is going to happen, because that wasn’t one day, or two days, it was almost six, seven months smelling that odor, and how can I erase that record. I live here, and I am going to be affected” (FG5 P6, translated from Spanish).

Participants described being told that there would be no long-term health effects, but a few participants report still experiencing lasting health symptoms over a year after the worst odors were experienced. One white woman explained,

I woke up the morning that it happened, and I had a headache that was one of the worst headaches I’ve ever had. I still have headaches to this day. I have not had a single day without a headache since this all started. (FG2 P2)

### 4) “It was really very much a grassroots effort”: Efforts to Build Community Power

Residents sought to take control of the crisis through information sharing and community networking, activism, and self-research.

#### Information sharing

Because many participants did not receive any information about the odor event from official sources or they did not trust the information they received, most participants noted that they got information about the incident from neighbors, Facebook groups, NextDoor posts, homeowners associations (HOAs), local community organizations, and active citizens who became community leaders during the event. Residents in turn shared information they received through their social networks. Some participants explained how they printed information they found online about the incident and H_2_S exposure and then posted the information for their neighbors who were not on social media. Other residents discovered resources that were available to them through informal networks that shared knowledge and advice. For example, many residents heard from neighbors and impromptu community leaders about the air purifiers and hotel reimbursements provided by the county. However, this information still missed some communities. While the majority of participants in the English focus groups received air purifiers, the only participant in the Spanish focus groups to receive an air filter found out about the program through information passed along to her husband at his work. Participants in the Spanish focus groups seemed to obtain information somewhat differently from the English-speaking participants, with most Spanish-speaking participants unaware of the community Facebook groups where residents shared information and many obtaining information directly from neighbors and HOA meetings.

#### Activism

Neighbors and community groups came together during the odor crisis to put pressure on local leaders. Several participants felt that there would have been no response to the odor crisis without impromptu community leaders stepping up demanding action and residents constantly complaining. Participants described the “marches and protests right outside of city hall” and how all emergency response was only a reaction to activism (FG4 P6). One Black woman described:

Nothing would have happened, absolutely nothing, because everything was a response to them. So everything was a response to all the grassroots. People who were trying to give the information, who are fighting….They do Zoom meetings, and they’d invite us to the Zoom meetings to talk. It was a lot because we were all involved in it, because we were all affected by it, and we all wanted answers. (FG2 P3)

Participants’ frustration with the local government’s emergency response contrasted with participants’ gratitude and pride of the grassroots response. One woman expressed, “I feel like the neighbors were what really helped….I don’t feel like the city slash government or county did a very good job at all” (FG2 P2). Residents also noted that they felt closer to their neighbors and “way connected to my community at that time” of the odor event (FG4 P1).

#### Self-research

Because residents were not getting information or did not believe the information they received, some participants described how they “were doing our own research” (FG3 P1). This self-research typically involved searching the internet for information on rotten egg smells and hydrogen sulfide. While only a few participants explained that they conducted their own research through online searches, these participants often shared their “self-researched” information with neighbors and friends.

### 5) “Maybe it’s toxic all the time?”: Long-term impacts of event

#### “I lost faith in our city as a government entity”: Event reinforced a lack of trust in the government and responsible agencies

Several participants expressed that they expected a much better response from the local government during this emergency, but many said that the perceived poor response only reinforced their lack of trust in the government. One Latina participant stated, “You know how politicians are, they all cover the truth so that one, the people don’t know so much about what is happening” (FG5 P6). Most participants agreed that the City of Carson and responsible agencies were not being transparent about the event and its effects; some people believe the city was purposely withholding information, lying or tricking residents, and “try[ing] to cover it up” (FG1 P2). One white woman described how mixed communication from the local government added to her stress and distrust:

At first, they were just saying placating things. Like, oh, no, it’s not the fire, it’s not this, it’s not that. Then when they did the research, yes, it was. I mean, that was one part of it. So that gave me a certain amount of anxiety and just awareness, like how much can we trust the people that are our politicians that can help us keep a healthy, clean environment? (FG3 P2)

Several participants expressed frustration that they felt the responsible agencies did not give consistent and clear information. A Black woman explained:

“I would say I don’t trust them either because they tell you what they want you to know. They don’t tell the truth.” (FG4 P4)

Participants were also frustrated by what they considered a late and incomprehensive response from the government. Even when the city and the county began distributing air purifiers and reimbursing people for hotel rooms and purifiers, participants expressed financial stress because of difficulties with the reimbursement process. Participants described that they often had to pay more money than would be reimbursed. Additionally, participants were frustrated with the small size of the air purifiers, and many people felt that they were ineffective; only residents with certain sized homes were given two purifiers.

#### “Absolutely, I think it’s a class issue”: Environmental justice and power of industry

Participants believe that the response would have been much quicker if this odor event occurred in an affluent area. While a few participants thought racism played a role in this event, most agreed that the injustice was related to class and socioeconomic status. A Latino participant expressed:

I want to agree that it’s definitely a class issue…the industry holds more power in these areas, including Long Beach and Carson….I just think the refineries have a lot of power because of how much money they produce for the city, and I think they’re able to like, hush the city, in a sense. So, it also has to do with that and it’s definitely power over industry than people, because at the end of the day the money stream is coming from that source, and it’s very unfortunate...I always thought that industry was the issue, but I thought I was just exaggerating...This reassured me that I’m not exaggerating…they’re putting lives at risk for what, you know. So, it just reaffirmed. (FG2 P1)

Multiple participants commented on how industrial interests are prioritized over health in the Carson area because of industry money. Participants also expressed worry about how these unjust exposures affect children, older people, and people with asthma and existing health conditions.

#### “I take notes of how it smells outside now”: Increased awareness of odors and pollution

The odor event prompted some participants to think more critically about their environment. One man explained, “I never really thought about Carson. It really has a lot of places where it’s all industrial…and the refineries are just right there, too” (FG1 P5). Many participants described that this chemical emergency increased their awareness of odors and pollution. A Black man explained:

We all [are] pretty much probably aware of our surrounding more special when it comes to the air because we know what happened to us….It definitely has you on guard because any time now, you know, like you smell something that’s, you know, foul in the air or anything, that’s out of norm, you know, flag raise up or what’s that? You know, how long is this gonna go on? (FG1 P2)

Focus group participants expressed concerns about various environmental exposures, including trucks, refineries, contaminated water and soil, and “Carson’s history of dump sites.” Several people also noted the historical neglect of the Carson area, resulting in industrial pollution and the limitations of the built environment.

## Discussion

We conducted focus groups to understand experiences of Carson residents during the 2021 hydrogen sulfide crisis, particularly regarding information acquisition, impressions of the emergency response, and immediate and ongoing physical and mental health symptoms. We identified five main themes from the focus group discussions: 1) difficulty in obtaining clear information about the event which led to feelings of abandonment, 2) responsible agencies downplaying residents’ concerns which resulted in residents feeling gaslit, 3) stress from the unknown health effects of the odors and continued stress/fear of future odor events, 4) efforts to build community power, and 5) lasting impacts of the event, including increased awareness of pollution and reduced trust in local agencies.

Although odors are a very common environmental concern, few studies have examined how sudden odor events affect residents [45]. The themes identified through our research are consistent with themes expressed by residents dealing with chronic environmental contamination and environmental disasters. Previous qualitative papers have described how residents facing environmental contamination experience anxiety, stress, worry, anger, and fear—emotions also described by participants in this study [26, 28, 47]. A study of the impact of a chemical contamination incident in a village in United Kingdom identified that lack of clear information led to rumors and increased anxiety among residents [26]. Several studies describe how ongoing uncertainty about the extent of the issue and lack of personal control over contamination events contribute to residents’ stress [26, 29, 34]. Much of the psychological stress during toxic exposure comes from the mixture of what is *known* (or believed to be true) about the environmental exposure and what is *unknown* and uncertain about the situation [27]. Environmental contamination physically harms the health of the residents, but the idea of the toxins is also harmful for mental health [28, 48]. Additionally, malodor has been associated with increased stress, irritation, annoyance, and poor mental health, as odor is an environmental stressor [35, 38, 49–53].

Residents affected by environmental contamination often describe receiving confusing, inadequate, ambiguous, or contractionary information [26, 28, 34]. Sometimes these confusing statements are based on conflicting research and existing epidemiological uncertainty, but poor communication and lack of transparency from responsible agencies has been found to exacerbate distress that residents already feel because of the toxins [26, 28]. A study of wellbeing among residents affected by per- and poly-fluoroalkyl substances (PFAS) contamination assessed how contamination is experienced at multiple geographical scales, including the body, home, local environment, and state [28]. These residents felt ignored, betrayed, abandoned, and disillusioned as they experienced the government downplaying the contamination [28]. PFAS exposed residents in the study described how their relationship with the government was permanently damaged because of their negative interactions regarding the contamination. The experiences and feelings of frustration, abandonment, and dismissal expressed by participants affected by contamination in these previous studies are very similar to those described by H_2_S-exposed participants in our Carson study.

Most of the themes we identified in this study are part of theoretical models or classification systems developed on the effects of toxic exposure. In a review that proposes a typology of psychosocial responses to environmental incidents, Page et al. separates the individual and societal impacts of contamination and describes lack of trust in authority as a way in which environmental contamination influences society [48]. A review of psychosocial health consequences of chronic environmental contamination divided risk factors into the material dimension (i.e., direct effects on health and environment) and the social dimension (i.e., community and institutional response to the environmental pollution/incident), which are typically correlated [29]. Sullivan et al. describes institutional delegitimization as the feeling that responsible institutions and healthcare providers are dismissing concerns about contamination-related health symptoms. Residents who experience health effects and institutional delegitimization during environmental contamination are at higher risk of psychological stress [29]. Institutional delegitimization was a particularly apparent theme in our focus groups on the 2021 H_2_S crisis. Additionally, other studies have identified similar lasting impacts of environmental contamination, including lack of trust in responsible agencies and increased awareness of pollution, further confirming the long-term effects of a pollution emergency that we observe [26, 48].

Most qualitative research on environmental contamination examines the impacts of invisible and odorless exposures, but smelling a toxin can greatly change one’s experience with contamination. Odor experiences can affect the sensitivity and acuity of olfaction [26, 54], and these H_2_S events may have increased residents’ long-term sensitivity to H_2_S and odor-related stress and health issues. Additionally, malodors often impact low-income communities, as they are often produced by industries (including refineries, rendering plants, pellet plants, industrial animal operations, hazardous waste sites, and landfills) that are disproportionately located near communities of color and in low-income areas [32, 55–62]. Malodors are a commonly ignored environmental justice issue, partly because odor is seen as subjective and is poorly regulated [31, 63]. Participants in our focus groups experienced the slow response to the H_2_S emergency as a form of injustice, which was compounded by their perception that government leaders referred to the odor as harmless and completely dismissed their concerns and health symptoms.

This H_2_S crisis disproportionately affected people of color and low-income residents in southern Los Angeles County [25]. In general, residents in environmental justice neighborhoods in the Carson area were inequitably exposed to H_2_S, disproportionately suffered from related health effects, and may be particularly sensitive to exposure to H_2_S and other air pollutants because of longstanding vulnerability from environmental and social stressors [25, 64]. People living in environmental justice communities disproportionately lack insurance, struggle to access hospital care, have pre-existing conditions, and lack resources to cope with abrupt environmental crises [65]. In the face of uncertain, strong malodors and related health symptoms experienced throughout the community, several residents rose to the occasion and suddenly became community leaders and activists organizing around eliminating the odors. Environmental justice community leaders have frequently been people new to activism who were spurred to organize because of sudden threats to their community’s health and wellbeing [14]. The Environmental Justice Movement consists of many local grassroots activists fighting for power, quality of life, and control over their own environment, as we also observed among impromptu activists in Carson during this H_2_S crisis [14].

While main themes of the focus groups conducted in English and Spanish were similar, we noticed that participants in the Spanish-speaking focus groups expressed stronger feelings of powerlessness, feelings of being forced to adapt to odors, and different information acquisition. A qualitative study of Latina mothers in South-Central and East Los Angeles–near Carson–identified foul odors as a main environmental concern, and they described their powerlessness, fear, uncertainty, and frustration around harmful environmental contaminants [66]. Communities often acquire information differently and receiving no information about an event that residents experience as an odor disaster can contribute to feelings of powerlessness.

Participants in our focus groups described receiving little or no information from local agencies and receiving most of their information from neighbors and social media. As many people rely on social media for news and notifications these days, emergency information needs to be dispersed using various sources, especially via social media. Although local agencies, including the South Coast Air Quality Management District, LA County Public Works, the City of Carson, have social media accounts, focus group participants predominantly obtained information on social media from friends, neighbors, and local Facebook groups, suggesting that the official emergency response may have benefited from a stronger social media presence. Research suggests that residents are more likely to follow emergency guidance when they hear it through family or friends or through social media than through radio, television, or public warning systems [67]; this highlights the importance of local government and emergency response teams partnering with communities to more effectively convey risk assessments and emergency instructions.

This study is among the first to assess residents’ experiences and perceptions during an odor emergency. The results may not be generalizable to the entire Carson community, as our study is limited by a small convenience sample of focus group participants that are predominantly women and Latinx. However, similar themes appeared in each focus group, and we conducted focus groups in both English and Spanish to understand the experiences of a diverse set of Carson residents. Additionally, we worked with community partners to refine the focus group guide, and we confirmed the themes we identified with several focus group participants to involve affected community members in this qualitative research.

## Conclusion

Community-engaged, qualitative research methods enabled us to better understand residents’ experiences and perceptions during this H_2_S emergency in Carson. Our study demonstrates the importance of clear, comprehensive, and timely responses to odor emergencies for addressing residents’ fears, curbing the spread of misinformation, and minimizing adverse health effects. Positive exchanges with responsible agencies, government responders, and healthcare providers have been found to reduce contamination-related stress [68]. The negative secondary social effects of contamination can be prevented when local leaders and public health professionals legitimize the potential stress of toxic exposure and effectively and transparently communicate risk [29]. Chemical emergencies can impact residents’ mental and physical health, and government and emergency response leaders can mitigate these health effects and feelings of institutional delegitimization by establishing genuine partnerships with community groups to assess needs, quickly developing interventions, and disseminating consistent and comprehensible information while considering equity issues. As environmental justice issues often lead to disaster vulnerabilities [16], just and equitable disaster preparedness, response, and recovery activities are needed in disaster-prone environmental justice communities.

### Supplementary Information

**Supplementary Material 1. **

## Data Availability

Focus group transcripts may include identifiable information about the participants and are not available to the general public. These transcripts may be available to interested researchers with the appropriate data use agreement.
