# Supplementary material for: Residents’ experiences during a hydrogen sulfide crisis in Carson, California
Source: Environ Health. 2024 Mar 23;23:31. doi: 10.1186/s12940-024-01071-5 (PMC10960400; doi:10.1186/s12940-024-01071-5)
Supplement: Supplementary file 1 — Supplementary Material 1. [file 12940_2024_1071_MOESM1_ESM.docx]

Supplementary Material for Quist et al. Residents’ experiences during a hydrogen sulfide crisis in Carson, California

Page 2: Focus Group Guide

Page 6: Original Spanish Quotes and English Translations

**Focus Group Guide**

***Before Focus Group begins:***

- *Participants read and sign informed consent form*
- *Participants complete basic demographic forms*

***Introduction:***

Welcome to the focus group on experiences with the Dominguez Channel Incident, odors, and environmental health concerns. This focus group is for research purposes, but we also hope it will be educational for you all as everyone shares their concerns and what kind of actions you all have taken or what actions you wish had been taken during the incident. Thank you for your time and for agreeing to be part of this focus group.

As a reminder, we are researchers at USC and Occidental College and we want to understand your experiences of the Dominguez Channel event any concerns that you have, especially about the environment and health in your community. We want to learn about your interests, concerns, and questions about the odor event, and its impact in the short and longer term for you, your family, and community. We will refer to the Dominguez Channel odor event, when thousands of residents in the Carson area experienced strong smells as “the odor event,” which took place in October-December 2021, although the time period during which you may have experienced smells may depend on where you live.

We expect for the focus group to last about 1-1.5 hours. I have a set of questions that I will use to guide us through the discussion. We want to recognize at the outset that we respect all viewpoints and appreciate if you can please ensure that everyone has a chance to speak, and that you wait until the person before you is done before speaking. For the first question, we will go around the room to hear each of your thoughts, but the majority of the session will flow like a conversation, and you should feel comfortable speaking up whenever you have something to share.

We are having a few different focus groups so after they are all completed, we will provide back the information we learned from all the groups together to you, so you have a sense of the broader range of community ideas and concerns. We hope to work with you to think about ways to protect public health in your neighborhoods.

This discussion will be tape recorded—if everyone agrees—so that we can check our notes to be certain we have heard your comments correctly. These recordings are for our internal purposes only. These recordings will be destroyed as soon as the analyses of the focus groups are completed.

Let’s go over a few ground rules that are helpful in keeping these discussions fun and productive.

- Please let me know if you do not understand a question.
- Only one person should speak at a time.
- We want to hear from everyone. Everyone’s views are important. Let’s have an open conversation today, so that people are comfortable saying whatever they feel.
- There are no right or wrong answers. Please give us your honest opinions. We are here to learn from you. Please feel free to share your point of view even if it differs from what others have said.
- Feel free to leave to use the rest rooms. [TELL THEM WHERE THE RESTROOMS ARE] Feel free to eat during our discussion.
- Please silence your cell phone.

Does anyone have any questions before we begin? Okay. Let’s get started.

**Carson Focus Group Questions**

***Icebreaker***

- First, I’d like to go around and have everyone introduce themselves. Tell us your name, approximately how far you live from the Dominguez Channel, and how long you have lived in the Carson area.

***Topic 1: Dominguez Channel Incident (45 minutes)***

Thank you for sharing. We will go around once more, and I’d like everyone to briefly describe your experience of the odor incident and share anything you’d like about the odors. How do you think the odor event affected you and your family, including your quality of life, physical and mental health, and relationships?

- How have the odors affected your daily stress or anxiety? How have you noticed stress or worries from the odors interacting with other stressors in your life?
- What did you think was causing the odor when you first smelled it?

*Individual and community coping mechanisms and support*

- Can you tell us a bit about your response to the event? Did you use any **coping mechanisms** or strategies during the odor event? What were these? And do you feel they helped? How did they help or what relief did they provide?
- Did you receive any **support** from neighbors, community, and/or non-profit organizations? What types of support did you receive or give?
  - Probing questions:
    - Did you become involved or desired to become involved in community initiatives?
    - Did the support that you gave or receive help you feel **connected** to your community? Or more disconnected?

*Information Sources and Regulatory Responses*

- Thinking back to the odor incident, how did you get **information** during the incident?
  - Probing questions:
    - What information did you receive from a government, environmental, or public health agency about the Dominguez Channel odor event?
    - Did you seek information or treatment from a medical professional?
- How did the information you receive during the odor event affect your **decisions** (e.g., whether to relocate, to open or close windows, to spend time outside or inside, etc.)?
  - Probing questions:
    - Did you change any behaviors during the event, if so, why?
    - Did you hear of or use any health resources?
- How do you feel about the way **local government** agencies responded to the odor event?
  - Probing questions:
    - Did you interact with any government agencies? Which one(s), what did they provide, and what were your experiences with them?
      - Examples: Department of Public Works, Department of Public Health, and AQMD (Air Quality Management District)
    - Were you **satisfied** with the resources and information that were provided?
    - Has the event affected your thoughts about local leadership and regulatory agencies? Did you ever consider these agencies before and if so, how? Did anything change?
      - Probing question:
        - Do you trust your local agency? If so, in what ways? Did this change at all as a result of this incident?

*Lasting perceptions of event*

- How have your **perceptions** and worries about odors, air pollution, industrial hazards, and/or hydrogen sulfide issues changed over the past year?
  - Probing questions:
    - How do you perceive the malodors; do they feel like a nuisance, a health threat, a stressor?
    - Did the event contribute to feelings of injustice or inequity? How satisfied are you with how the event what handled? Do you feel it is ongoing or has it concluded?

***Topic 2: Learning about community and environmental concerns: (10 minutes)***

Now we are going to talk about your experiences in your community and environment more generally.

- What are your main concerns and worries about your community and the physical environment?
  - Probing questions:
    - What do you feel are the most urgent issues in your community?
    - What kind of changes would you like to see in your environment and community?
    - Are you currently concerned about odors, air pollution, extreme heat, or certain industries?
      - This could include concerns about trucks, traffic, oil and gas drilling, refineries, rendering plants, industrial chemicals, industrial fires/explosions, sewage, landfills, unclean water, extreme heat, pesticides, etc.
    - Are your concerns based on worries regarding health, inequity, nuisance, etc.?
- What kind of information would you like to see about pollution and environmental hazards?
- To end on a positive note, what do you enjoy and appreciate most about your community and the physical environment?

Those are all the questions I have.

***Wrap up: 5 minutes***

- Is there anything that I neglected to ask about that you think is important regarding Dominguez Channel odor incident and health and environmental concerns? Or is there anything you would like to add to what has been said?

This has been very helpful. Thank you very much.

**Original Spanish Quotes and English Translations:**

**Spanish**: “yo llamé por teléfono varias veces a un número que me dieron cuando vinieron aquí y este y marcaba y muchas veces no contestaban.”

**English**: “I called a few different times to the number they gave me when they came here [HOA meeting] and I kept calling but they never answered.” (Focus group 5, Participant 5)

**Spanish**: “Pues sí. Pues sí, en esta area estamos olvidados.”

**English**: “Well yeah, in this area we are forgotten.” (FG6 P3)

**Spanish**: “Si esta área, esta área tiene menos recursos, es 90810.”

**English**: “Yeah this area, this area has less resources, its 90810” (FG5 P6)

**Spanish**: “Y como nuestro código postal coincide con Long Beach 90810, nosotros nos dicen que nosotros somos parte de Long Beach, no somos parte de Carson y es por eso que los recursos para Carson con nosotros no se vienen Es algo que nosotros hemos estado peleando.....cuando embellecen Carson, embellecen de la Avalon para adelante. Todo eso está bien embellecido, Carson Pero esa parte, la olvidada, apenas nos pusieron los crossing guard y toda la cosa.”

**English**: “And because our postal code shows up as Long Beach, they tell us we are a part of Long Beach and not Carson, and that’s why we don’t get any resources. It is something that we have been fighting against....When they beautify Carson, they improve the Avalon area. All of that area is improved, but this part, the forgotten part, they barely gave us a crossing guard and all that.” (FG5 P7)

**Spanish**: "Y no sabemos a quién dirigirnos. Quizá porque estamos más lejos y el centro de Carson está allá.”

**English**: “we don’t know who to go to. Maybe because we are farther from the center of Carson over there.” (FG6 P1)

**Spanish**: “Sí, Pues no hemos tenido ningún apoyo, ninguna ayuda ni nada.”

**English**: "We have not had any support, no help, nothing.” (FG5 P5)

**Spanish**: “Dices solo que no está en mis manos de solucionarlo, entonces aprendo a convivir con él, porque pues no, no se podía hacer nada porque ya estaba trabajando por aquí cerca y pues ni modo, aunque estuvieras sofocado porque no te gustaba lo que estaba sintiendo diferente, pero tenías que seguir con tus labores, no podías detenerte entonces, como que adaptas la situación.”

**English**: “You tell yourself, it’s not in my hands to solve the problem, so you just have to learn how to live with it. You can’t do anything because your job is right there, and even if you are suffocating and you don’t like how you are feeling, you have to continue with your work you can’t just quit your job. So that’s why I think we had to adapt to the situation.” (FG6 P2)

**Spanish**: “Y ese es el miedo que uno como residente de Carson, de que tal vez en este momento no tenemos ninguna secuela, ninguna enfermedad. Pero tal vez en un futuro se puede desarrollar un cáncer en los pulmones o algo que vaya a suceder, porque eso no fue de uno un día, dos días, fue casi como seis, siete meses oliendo ese olor y pues como borrar ese registro, como decir oh yo vivo aqui y yo voy a ser afectada.”

**English**: “And that is the fear of being a Carson resident, that maybe in this moment we don’t have any condition, or any sickness. Buy maybe in the future you can develop lung cancer or something that is going to happen, because that wasn’t one day, or two days, it was almost six, seven months smelling that odor, and how can I erase that record. I live here, and I am going to be affected.” (FG5 P6)

**Spanish**: “ya sabes cómo son los políticos, todo lo cubren verdad para que uno, el pueblo no sepa tanto qué es lo que ocurre.”

**English**: “You know how politicians are, they all cover the truth so that one, the people don’t know so much about what is happening.” (FG5 P6)
